# SARS CoV-2 Organotropism Associated Pathogenic Relationship of Gut-Brain Axis and Illness

**DOI:** 10.3389/fmolb.2020.606779

**Published:** 2020-12-22

**Authors:** Pottathil Shinu, Mohamed A. Morsy, Pran Kishore Deb, Anroop B. Nair, Manoj Goyal, Jigar Shah, Sabna Kotta

**Affiliations:** ^1^Department of Biomedical Sciences, College of Clinical Pharmacy, King Faisal University, Al-Ahsa, Saudi Arabia; ^2^Department of Pharmaceutical Sciences, College of Clinical Pharmacy, King Faisal University, Al-Ahsa, Saudi Arabia; ^3^Department of Pharmacology, Faculty of Medicine, Minia University, El-Minia, Egypt; ^4^Department of Pharmaceutical Sciences, Faculty of Pharmacy, Philadelphia University, Amman, Jordan; ^5^Department of Anesthesia Technology, College of Applied Medical Sciences in Jubail, Imam Abdulrahman bin Faisal University, Dammam, Saudi Arabia; ^6^Department of Pharmaceutics, Institute of Pharmacy, Nirma University, Ahmedabad, India; ^7^Department of Pharmaceutics, Faculty of Pharmacy, King Abdulaziz University, Jeddah, Saudi Arabia

**Keywords:** SARS-CoV-2, COVID-19 infection, gut-brain axis, ACE2 receptor, microbiota, dysbiosis, cytokine storm

## Abstract

COVID-19 has resulted in a pandemic after its first appearance in a pneumonia patient in China in early December 2019. As per WHO, this global outbreak of novel COVID-19 has resulted in 28,329,790 laboratory-confirmed cases and 911,877 deaths which have been reported from 210 countries as on 12^th^ Sep 2020. The major symptoms at the beginning of COVID-19 are fever (98%), tussis (76%), sore throat (17%), rhinorrhea (2%), chest pain (2%), and myalgia or fatigue (44%). Furthermore, acute respiratory distress syndrome (61.1%), cardiac dysrhythmia (44.4%), shock (30.6%), hemoptysis (5%), stroke (5%), acute cardiac injury (12%), acute kidney injury (36.6%), dermatological symptoms with maculopapular exanthema (36.1%), and death can occur in severe cases. Even though human coronavirus (CoV) is mainly responsible for the infections of the respiratory tract, some studies have shown CoV (in case of Severe Acute Respiratory Syndrome, SARS and Middle East Respiratory Syndrome, MERS) to possess potential to spread to extra-pulmonary organs including the nervous system as well as gastrointestinal tract (GIT). Patients infected with COVID-19 have also shown symptoms associated with neurological and enteric infection like disorders related to smell/taste, loss of appetite, nausea, emesis, diarrhea, and pain in the abdomen. In the present review, we attempt to evaluate the understanding of basic mechanisms involved in clinical manifestations of COVID-19, mainly focusing on interaction of COVID-19 with gut-brain axis. This review combines both biological characteristics of the virus and its clinical manifestations in order to comprehend an insight into the fundamental potential mechanisms of COVID-19 virus infection, and thus endorse in the advancement of prophylactic and treatment strategies.

## Introduction

There have been three outbreaks of human pathogenic coronavirus (CoV) in the 21^st^ century namely, severe acute respiratory syndrome CoV (SARS-CoV) in 2003, Middle East respiratory syndrome CoV (MERS-CoV) in 2012 and new CoV disease 2019 (COVID-19) (first reported in China in 2019), causing transmission globally, which has caused global public health problems and economic development related challenges (Stadler et al., 2003; Cui et al., 2019; Lu et al., 2020; Memish et al., 2020; Wu et al., 2020a; Zhou et al., 2020a). The COVID-19 pandemic has resulted in 28,329,790 laboratory-confirmed cases and 911,877 deaths across 210 countries as on 12^th^ Sep 2020, as per World Health Organization (WHO) (Organization and Organization, 2020). With the help of electron microscopy, it has been found that the virus possesses an envelope with round or oval particles called spikes (60–140 nm diameter) (Ahn et al., 2020; Fan et al., 2020). The genome sequences of novel CoV-2019 were related to SARS-CoV belonging to beta-CoV family and it also showed ~79% and ~50% similarity to SARS-CoV and MERS-CoV, respectively (Lu et al., 2020). Although SARS-CoV-2 is known to be infectious with a basic reproduction number (R_0_) of 3.77 (SARS, R_0_ = 3–5) (Li et al., 2020b; Yang et al., 2020b), most of the cases are mild and have a low mortality rate except among older patients (≥60 years of age) and patients with pre-existing comorbidities like hypertension, diabetes mellitus, and cardiovascular diseases (Baghizadeh Fini, 2020; Chen et al., 2020; Contini et al., 2020). It has been observed that many patients suffering from SARS CoV-2 infection are also associated with gastrointestinal and neurological symptoms. In SARS CoV infections, the intestinal inflammation is mainly modulated by the ACE2 receptor mediated mechanisms (that mainly regulate the gut-microbial flora) and the similar mechanism may be implicated in SARS CoV-2 infections as well (Yang et al., 2020a). Disturbances of gut-microbial flora may be a factor behind the CNS symptoms like confusion and delirium. The present review is an attempt to underpin the possible interplay between COVID-19, ACE2 and CNS and GIT symptoms.

## Selection of Literatures for Review

**Figure 1:**
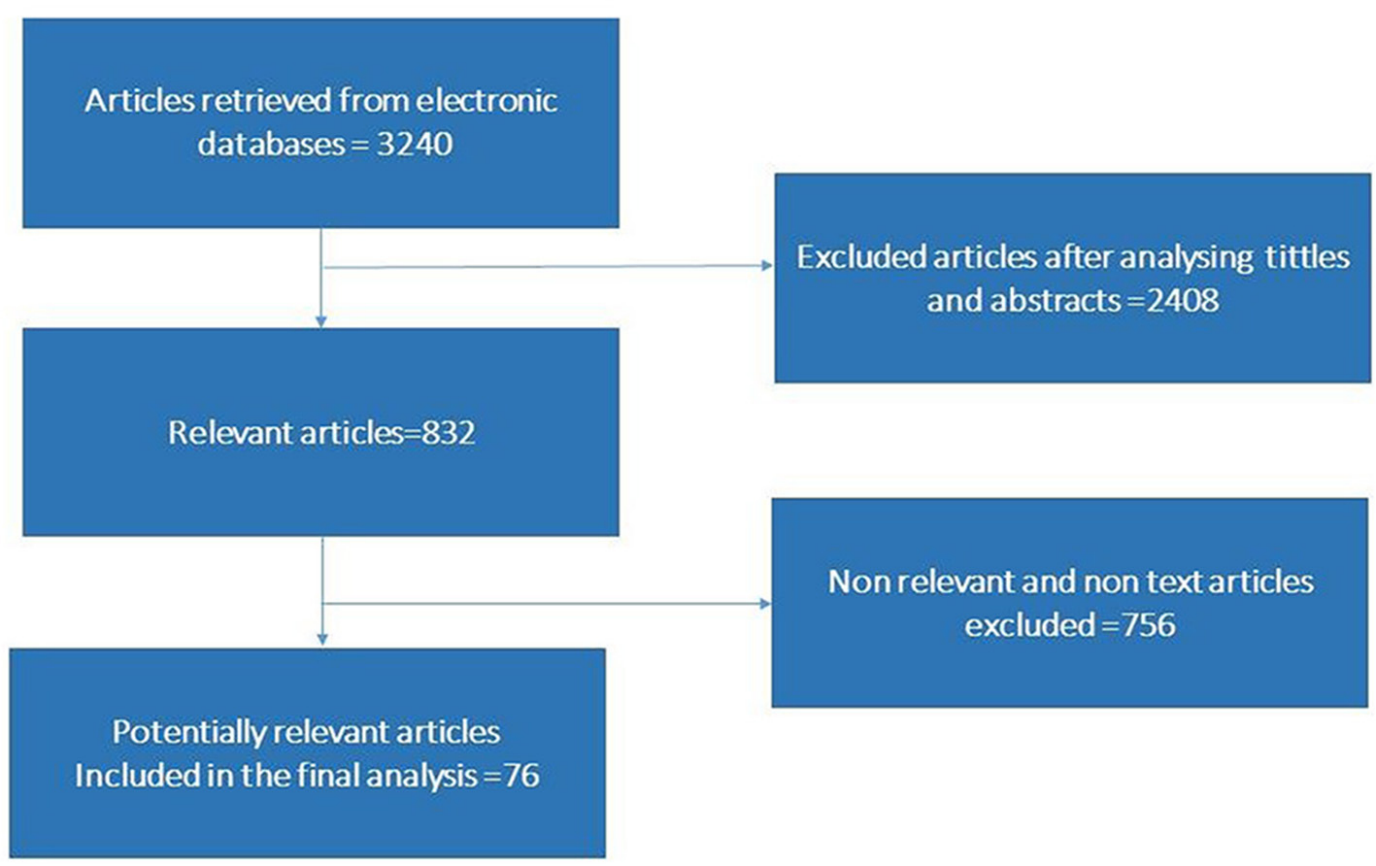
Flow chart showing article search and selection criteria.

Relevant studies were retrieved from Science Direct, Medline, Public Library of Science, Mendeley, PubMed, Springer Link, and Google Scholar. We used multiple keywords like “microbiota,” “microbiome,” “microbial communities,” “gut-microbiota,” “SARS-CoV-2 infection,” “COVID-19 infection,” “Pathogenesis of COVID-19 infection,” “SARS CoV-2 transmission,” “SARS-CoV-2 mediated gastrointestinal infection,” “COVID-19 infection associated psychotic problems,” “COVID-19 infection associated cerebrovascular changes,” “Relationship of gut microbiota with brain” or “COVID-19 mediated inflammatory response,” in combination with “Cytokine storm,” “Immunogenic profile,” “SARS-CoV-2 mediated dysbiosis and dysbiosis altered mRNA profile,” “Immunomodulatory response changes brain physiology” individually and in combination for literature search. The overall method of article screening and selection criteria are depicted in Figure 1. The articles in English language are used to compile the information. We also screened the reference list of the articles retrieved in order to find articles that remained unidentified by initial search strategy.

## COVID-19 Infection Associated Clinical Feature and Illness

**Figure 2:**
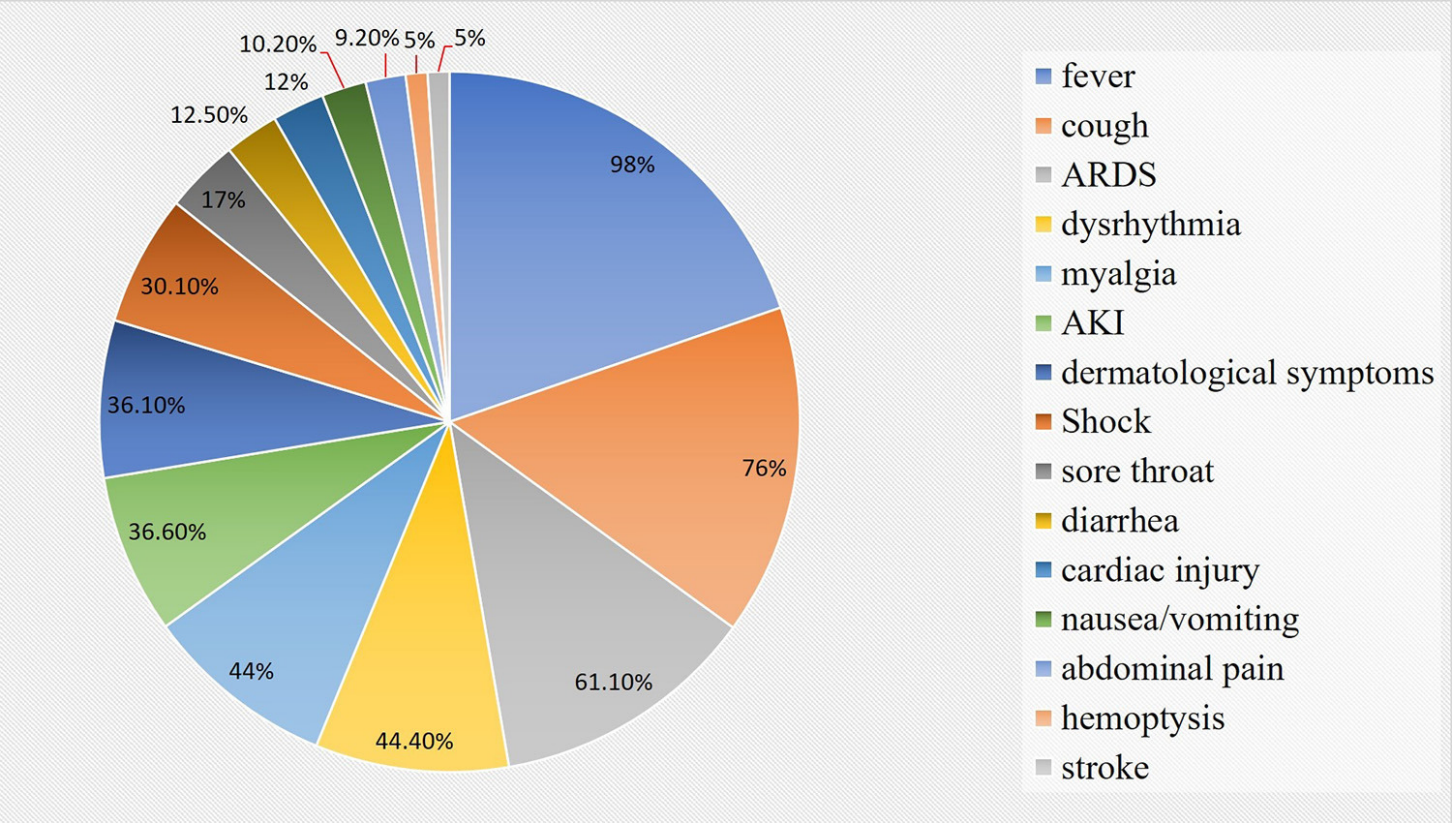
Pie chart data showing clinical manifestations of COVID-19 infection.

As per WHO, COVID-19 is the third episode of spillover of an animal CoV to humans in the last 20 years that has caused major epidemic (Yang et al., 2020b). The main route of transmission of SARS-CoV-2 is through aerosolized droplets (like other CoV), additionally, the infection may also be transmitted through direct physical contact, mother-to-child (García, 2020; Harapan et al., 2020). The nucleic acid of SARS-CoV-2 has also been found in fecal specimens that suggest the potentiality of the gastrointestinal tract (GIT) in the transmission, even though it requires further studies in order to consider it as an established fact (Holshue et al., 2020). The incubation period of SARS-CoV-2 continues to be 5–14 days as per a retrospective study (Lu et al., 2020); however, a more recent study suggests the incubation period to be 24 days (Izzetti et al., 2020). At the beginning of COVID-19 pandemic, the main symptoms were fever (98%), tussis (76%), sore throat (17%), rhinorrhea (2%), chest pain (2%), and myalgia or fatigue (44%). Further, acute respiratory distress syndrome (61.1%), cardiac dysrhythmia (44.4%), shock (30.6%), hemoptysis (5%), stroke (5%), acute cardiac injury (12%), acute kidney injury (36.6%), dermatological symptoms with maculopapular exanthema (36.1%) and death can occur in severe cases (Figure 2) (Jiang et al., 2020; Kandeel and Al-Nazawi, 2020).

Although human CoV leads to infections of the respiratory tract, some studies have also demonstrated its potentiality to spread into extra-pulmonary organs including the nervous system and the GIT, as observed in cases of SARS and MERS (Kannan et al., 2020). As per recent reports, COVID-19 patients have also presented with neurological and enteric infection related symptoms such as disorders related to smell/taste, loss of appetite, nausea, emesis, pain in the abdomen, and diarrhea (Kotwani and Gandra, 2020; Lake, 2020).

## SARS-CoV-2 Organotropism and Mechanism of Transmission

Pharmacologically, enough evidence has been collected to establish the involvement of several important pathological molecular mechanisms in the spread of SARS-CoV-2 via aerosolized droplets into the lungs. The genome sequence of SARS-CoV-2 consists of 29 kb bases and 12 protein-coding regions (1ab, 1a, S, 3a, 4, M, 6, 7a, 7b, 8, N, 10) (Walls et al., 2020). Spike (S) protein mediates the entry of SARS-CoV-2 into the host cells. This protein comprises of two functional subunits–S1 (for attaching with the host cell) and S2 (helps in the fusion with the cellular membranes) (Li et al., 2020,a). The protein S of many CoVs are separated at the border between S1 and S2 subunits, which stay bound non-covalently in prefusion conformation (Walls et al., 2020). The S1 subunit (distal) carries receptor-binding domain and helps in stabilizing the prefusion state of membrane-anchored S2 subunit, which also acts as receptor for fusion (Liu et al., 2020a,b). The protein S for all CoVs is further cleaved by proteases of the host at the S2′ position, which is situated upstream to the fusion peptide. Hence, entry of CoV into the cells that are susceptible is a harmonious and complex process, which needs an intensive mechanism of binding of receptors and the proteolytic dispensation of the protein S that helps in the promotion of virus-cell fusion (Liu et al., 2020c). The most common entry mechanism of a virus into the host cell is via endocytosis, which is a receptor-mediated process. It is believed that the receptor, angiotensin-converting enzyme-2 (ACE2; protein found on surface of cells), and TMPRSS2 (a gene that encode for transmembrane protease enzyme and belong to serine protease family) are expressed in lung tissues and epithelial cells, which are employed by CoV to establish infection in the lungs (Ozma et al., 2020; Porfidia and Pola, 2020). There is also a known fact that ACE2 receptors are expressed on heart, esophageal, ileal, renal tissues at a level higher than that in the alveolar cells. This suggests the involvement of multi-organ systems in severe SARS-CoV-2 infection (Rico-Mesa et al., 2020). The virus binds with ACE2 receptors (present on the plasma membranes of the epithelial cells) of host cells particularly located on the respiratory tract and upper esophagus (Shanmugaraj et al., 2020). The SARS-CoV-2 can also infect cell types like stratified epithelial cells and enterocytes of colon and ileum. In addition, virus also infects cholangiocytes, proximal tubule cells of nephrons, urothelial cells of the urinary bladder, and myocardial cells of heart (Shen et al., 2020). It is known that the *in vitro* isolation of SARS-CoV-2 usually require 6 days in cell lines like Vero-E6 and Huh-7, but virus infects the epithelial cells lines within 96 h. However, the SARS-CoV-2 does not affect T cells, CD4 or ACE2 cells (Sifuentes-Rodríguez and Palacios-Reyes, 2020) and thus not damaging the immune systems. Moreover, nucleotide sequencing analysis revealed seven major genetic variations occurred in SARS CoV-2 indicating that the ongoing human infection may be a recent incident. These genetic variations may change the virus to remerge as more virulent to infect digestive, circulatory, urogenital, and central nervous system apart from respiratory tract infections (Hussain et al., 2020).

## Pathogenesis and Clinical Consequences in Gastrointestinal System During SARS-CoV-2 Attack

The clinical characteristics as well as the epidemiology of disorders related to GIT in COVID-19 patients has been analyzed by various researchers. Different studies report that various percentage of COVID-19 patients were affected by GIT related disorders like emesis, nausea, and diarrhea. In one study, a total of 651 COVID-19 patients enrolled before they were treated with antivirals or antibiotics and it was observed that 11.4% of the patients suffered from at least one common symptom related to the GIT namely diarrhea (Jin et al., 2020). In this study, the disorders related to intestines persisted for a median of 4 days and preceded symptoms related to the respiratory system. Symptoms related to GIT were comparatively higher among critically ill COVID-19 patients (23%) than in mild COVID-19 cases (8%) (Sohrabi et al., 2020). Several studies involving varying numbers of COVID-19 patients (*n* = 254, 59, 204, 58, 138, and 1099) suggested development of disorders related to the GIT in 26, 25.4, 18.6, 11, 13.7, and 8.7% of the COVID-19 patients, respectively (Borah et al., 2020; Cheung et al., 2020; Lin et al., 2020; Pan et al., 2020; Wang et al., 2020a; Zhou et al., 2020b). Other studies carried out in the United States and Europe enrolling varying number of patients (*n* = 278, 318, and 40) showed GIT related signs and symptoms in 35, 61, and 55% patients, respectively. The pooled data of prevalence of GIT symptoms including anorexia (26.8%), diarrhea (12.5%), nausea/emesis (10.2%), and pain/discomfort in the abdomen was seen in a meta-analysis study that included 4243 COVID-19 patients across 6 countries (Tian et al., 2020). SARS-CoV and SARS-CoV-2 have nearly 80% resemblance and hence, infections of GIT that mainly mediated through ACE2 receptors was expected. ACE2 receptors are expressed in enterocytes of the small intestine, and its rate of binding affinity influences the rate of infectivity of the virus. In addition, in comparison with the previous SARS-CoV outbreaks, SARS-CoV-2 RNA was detectable in the stool samples though it was not found in the respiratory tract (Velavan and Meyer, 2020).

In SARS-CoV infections, the intestinal inflammation is mainly modulated by ACE2 receptor mediated mechanism and therefore, its disruption by SARS-CoV-2 may result in diarrhea and other GIT related complications. Recruiting inflammatory cells from the bone marrow, systemic or local production of inflammatory cytokines like interleukin (IL)-6, tumor necrosis factor-alpha (TNF-α), IL-1β, 2,7,8,9,10,17, monocyte chemotactic protein 1 (MCP1), macrophage inflammatory protein 1A (MIP1A), macrophage inflammatory protein 1B (MIP1B), macrophage inflammatory protein 3A (MIP3A), elevated levels of procalcitonin, increased levels of ferritin d-dimer, C-reactive protein (CRP), and activating JAK-STAT protein may also contribute in the etiology of the GIT complications (Wang et al., 2020b; Wu et al., 2020b). Further, the excessive secretion of cytokines (cytokine storm) along the GIT wall may result in tissue damage. This cellular damage may also be induced by viral replication, which might impart in the development of injury and inflammation of the gut epithelium. These inflammatory cytokines stimulate the vagus nerve to promote nausea, vomiting, and diarrhea that usually occur in complicated cases of COVID-19 infections (Wu et al., 2020c). A recent study carried out on complicated cases of COVID-19 revealed that infection with SARS-CoV-2 could alter the gut microbiota. This study reveals relatively increased cases of opportunistic infections of GIT that mainly caused by *Streptococcus, Rothia, Veillonella, Erysipelothrix, Clostridium*, and *Actinomyces* species in COVID-19 patients (Xiao et al., 2020).

The ACE2 receptors in the intestines are known to act as co-receptors for the intake of the nutrients including amino acid (AA) from food. Apart from controlling dietary AA homeostasis, ACE2 helps in regulating the development of gut-microbiota and innate immunity mechanisms (Yang et al., 2020a). Earlier reports reveals that the expression of AA transporter protein B^0^AT1 was not present in the small intestine of ACE2 mutant mice. Due to lack of expression of B^0^AT1 proteins in the intestines, the serum concentration of non-essential AA valine (Val), threonine (Thr), and tyrosine (Tyr) and the essential AA tryptophan (Trp) were significantly decreased in *Ace2*^−/*y*^ mice (Ye et al., 2020). Further, these AAs (particularly Trp) acts as precursors for the synthesis of monoamine neurotransmitters serotonin [5-hydroxytyphtophan (5-HT)] and catecholamines in the brain. Furthermore, the rate of production of 5-HT and catecholamines was directly associated with their local substrate availability in the brain (Yu et al., 2020). This might further result in reduction in the production of 5-HT and catecholamines. Therefore, severe COVID-19 patients with GIT related complications might develop pathophysiological conditions like depression, delirium, and confusion.

## Altered Gut Activity Mediated Psychotic Changes in Severe COVID-19 Infection

**Figure 3:**
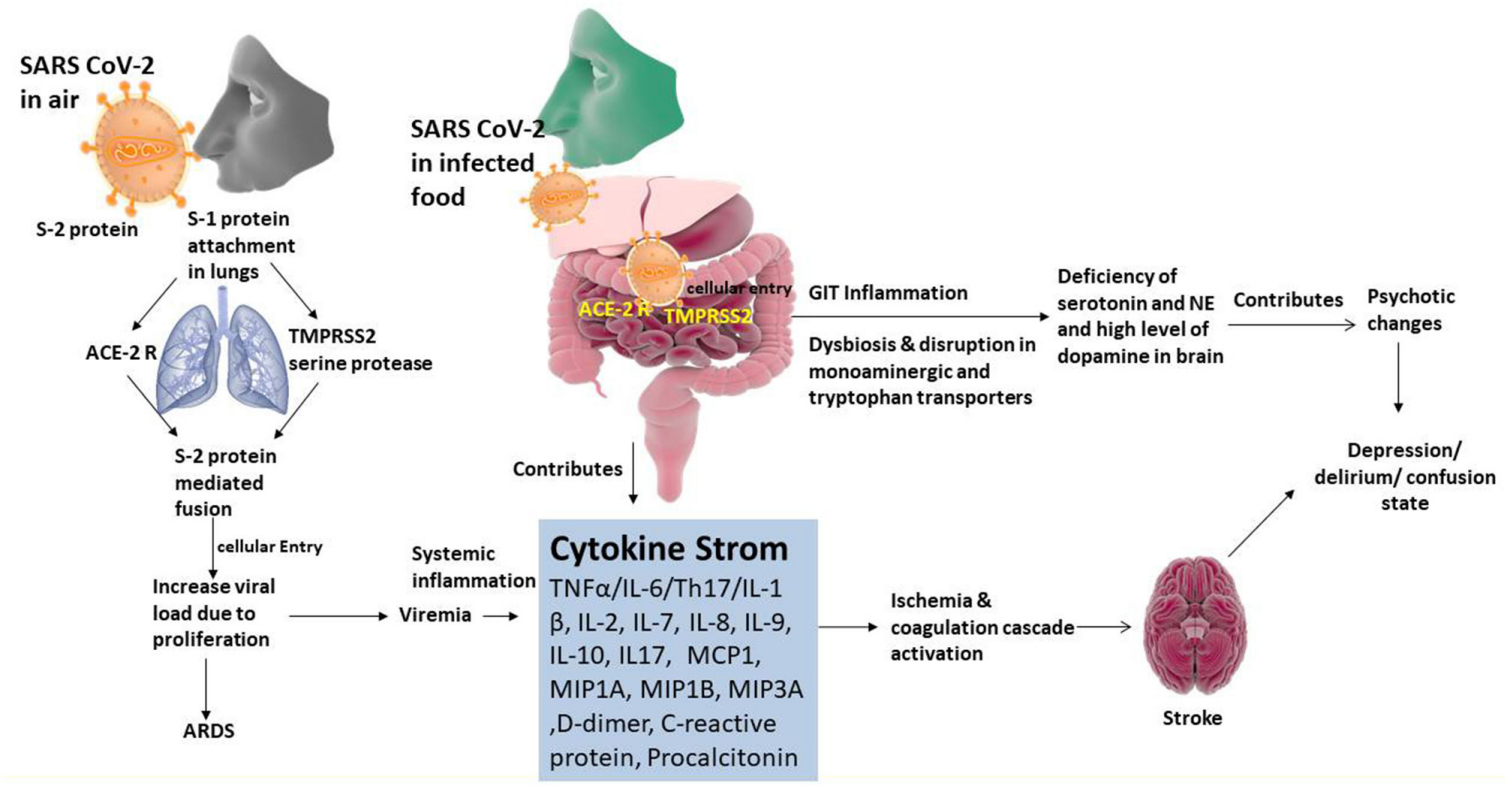
Organotropism of SARS CoV-2 associated pathogenic relationship of gut-brain axis and psychotic illness.

The gut-brain axis is a two-way system that connects cognitive centers of the brain with peripheral working of the digestive tract (Figure 3). It is evident that changes in gut microbiota may have influence on the development of behavioral changes like depression, delirium/confusion. This is in turn most likely associated with the rate of absorption of Trp from the GIT and production of 5-HT in the brain tissues. (Yu et al., 2020). The gut microbiota or its metabolic end products stimulates vagus nerve in order to transmit the impulses to the solitary nucleus that also function as primary GIT sensory relay station, and then to the most vital centers such as thalamus, hypothalamus, locus ceruleus, amygdala, and periaqueductal gray (Al Omran and Aziz, 2014). These electrical impulses produced in the vagus nerve (as a result of stimulation of gut microbiota) will have an effect on 5-HT concentration in the brain tissues of both rodents and humans (Ressler and Mayberg, 2007). The gut microbiota influence the various neurotransmitter levels by stimulating the central nervous system and the gut *via* the synthesis of metabolites (Galland, 2014; Evrensel and Ceylan, 2015). These metabolites are the end products of normal bactrial flora of the GIT, which include short-chain fatty acids (SCFAs), bile acids, choline metabolites, lactate, and vitamins. These metabolites can directly or indirectly regulate the production of neurotransmitters (Galland, 2014). Immune cells and inflammatory mediators play various functions in the gut-brain axis communication ranging from physiological role in sleep and memory to pathophysiological role in neuropsychiatric conditions (Caspani et al., 2018). Litratures indicate that the patients with depression usually possess high concentrations of pro-inflammatory cytokines (for instance; IL-6, and TNF-α) as compared with normal individuals (Dantzer et al., 2008; Dowlati et al., 2009). These pro-inflammatory cytokines in the GIT may stiumulate vagus nerve in order to modulate central stress circuitry followed by activation of hypothalamic–pituitary–adrenal axis (Sternberg, 2006). Further, it is also known that patients suffering from depression may have abundance of *Streptococcus, Rothia, Veillonella, Erysipelothrix, Clostridium*, and *Actinomyces* species in their gut microbiota (Amirkhanzadeh Barandouzi et al., 2020) and it is established that an increased growth of *Streptococcus* species would raise the levels of IL-6 and TNF-α in humans (Jiang et al., 2015). This fact has been further supported by various studies wherein higher concentrations of pro-inflammatory cytokines (such as IL-1β and IL-6) and low concentrations of anti-inflammatory cytokines (like IL-4 and IL-10) were detected in patients enduring with depression (Berk et al., 2013; Wong et al., 2016). Additionally, the intestinal concentrations of 5-HT are retained by the enterochromaffin cells that possess tryptophan hydroxylase and stimulated by the metabolites of the gut like SCFA and bile acids (Annweiler et al., 2020; Bobker and Robbins, 2020). Moreover, it has been assumed that a reduction in levels of Trp might result in a decrease of 5-HT, which may lead to delirium development (Gunther et al., 2008). An increase in the levels of dopamine and development of delirium are inter-linked. Also, dopamine is linked to various metabolic pathways and Ca^2+^ channels which result in a marked elevation in the dopamine under an impaired oxidative condition. The Ca^2+^ influx in the cells results in an elevated dopamine production which results in uncoupling of oxidative phosphorylation mainly occur in the mitochondria of the brain parenchymal cells (Calcagno et al., 2020). The result is an elevated production of metabolites of dopamine that are toxic along with a decreased ATP production, which inhibits the catechol-O-methyl transferase (COMT) activity, an important enzyme that aids dopamine synthesis and breakdown in the prefrontal cortex (Gunther et al., 2008; Maldonado, 2008; Kamholz, 2010).

On the other hand, recently, a study showed resistance of norepinephrine (NE) transporter (NET) knockout mice toward depression-like behavioral changes, which are stress induced, as well as expression of brain neurotrophin which are seen in normal mice. Hence, depression and delirium can be caused due to reduced levels of NE and elevated concentrations of dopamine (Coleman et al., 2020; Das et al., 2020). Besides, CRP may lead to the stimulation of the formation of reactive oxygen species (ROS) which result in blood brain barrier disruption, thereby causes delirium. As per studies, a higher occurrence of delirium in post-operative hip surgery patients is associated with higher concentrations of CRP and IL-6 (Beloosesky et al., 2004; Macdonald et al., 2007). A prospective study was conducted wherein they measured the inflammatory biomarkers procalcitonin, and CRP in patients ventilated mechanically (McGrane et al., 2011). An association was found between delirium, less coma-free days, elevated levels of CRP and procalcitonin, which implicated inflammation as a vital mechanism in delirium pathophysiology.

## Conclusion

In nutshell, there is a need to pay attention to patients presenting initial symptoms related to the digestive system particularly diarrhea during the diagnosis and management of SARS-CoV-2. In addition, on the occurrence of diarrhea or other related symptoms during treatment, the patient should receive a prompt integrative surveillance of psychotic results. Thus, the treatment regimen should include anti-diarrhea therapy along with the probiotics (necessary for reactivation of beneficial intestinal microbiota) that will help in reducing the psychotic consequences of the central nervous system. In addition to antiviral intervention, the treatment regimen should include rejuvenating electrolytes along with sufficient water intake. Further, future studies are required to obtain insights into association between SARS-CoV-2 and the gut-brain axis.

## Author Contributions

PS: conceptualization, writing—original draft, and funding acquisition. MM and AN: supervision and writing—review and editing. PD: conceptualization and writing—review and editing. MG, JS, and SK: writing—review and editing. All authors contributed to the article and approved the submitted version.

## Conflict of Interest

The authors declare that the research was conducted in the absence of any commercial or financial relationships that could be construed as a potential conflict of interest.

## References

[B1] Ahn D. G. Shin H. J. Kim M. H. Lee S. Kim H. S. Myoung J.. (2020). Current status of epidemiology, diagnosis, therapeutics, and vaccines for novel coronavirus disease 2019 (COVID-19). J. Microbiol. Biotechnol. 30, 313–324. 10.4014/jmb.2003.03011 32238757 PMC9728410

[B2] Al Omran Y. Aziz Q. (2014). The brain-gut axis in health and disease. Adv. Exp. Med. Biol. 817, 135–153. 10.1007/978-1-4939-0897-4_6 24997032

[B3] Amirkhanzadeh Barandouzi Z. Starkweather A. Henderson W. Gyamfi A. Cong X. (2020). Altered composition of gut microbiota in depression: a systematic review. Front. Psychiatry 11:541. 10.3389/fpsyt.2020.00541 32587537 PMC7299157

[B4] Annweiler C. Cao Z. Wu Y. Faucon E. Mouhat S. Kovacic H.. (2020). Counter-regulatory ‘Renin-Angiotensin’ system-based candidate drugs to treat COVID-19 diseases in SARS-CoV-2-infected patients. Infect. Disord. Drug Targets 20, 407–408. 10.2174/1871526520666200518073329 32418532

[B5] Baghizadeh Fini M. (2020). What dentists need to know about COVID-19. Oral Oncol. 105:104741. 10.1016/j.oraloncology.2020.104741 32380453 PMC7186204

[B6] Beloosesky Y. Grinblat J. Pirotsky A. Weiss A. Hendel D. (2004). Different C-reactive protein kinetics in post-operative hip-fractured geriatric patients with and without complications. Gerontology 50, 216–222. 10.1159/000078350 15258426

[B7] Berk M. Williams L. J. Jacka F. N. O'neil A. Pasco J. A. Moylan S.. (2013). So depression is an inflammatory disease, but where does the inflammation come from? BMC Med. 11:200. 10.1186/1741-7015-11-200 24228900 PMC3846682

[B8] Bobker S. M. Robbins M. S. (2020). COVID-19 and headache: a primer for trainees. Headache 60, 1806–1811. 10.1111/head.13884 32521039 PMC7300928

[B9] Borah P. Deb P. K. Deka S. Venugopala K. N. Singh V. Mailavaram R. P.. (2020). Current scenario and future prospect in the management of COVID-19. Curr. Med. Chem. 10.2174/0929867327666200908113642 32900341

[B10] Calcagno N. Colombo E. Maranzano A. Pasquini J. Keller Sarmiento I. J. Trogu F.. (2020). Rising evidence for neurological involvement in COVID-19 pandemic. Neurol. Sci. 41, 1339–1341. 10.1007/s10072-020-04447-w 32394275 PMC7214096

[B11] Caspani G. Corbet Burcher G. Garralda M. E. Cooper M. Pierce C. M. Als L. C.. (2018). Inflammation and psychopathology in children following PICU admission: an exploratory study. Evid. Based Ment. Health 21, 139–144. 10.1136/ebmental-2018-300027 30301824 PMC6241628

[B12] Chen H. Guo J. Wang C. Luo F. Yu X. Zhang W.. (2020). Clinical characteristics and intrauterine vertical transmission potential of COVID-19 infection in nine pregnant women: a retrospective review of medical records. Lancet 395, 809–815. 10.1016/S0140-6736(20)30360-3 32151335 PMC7159281

[B13] Cheung K. S. Hung I. F. N. Chan P. P. Y. Lung K. C. Tso E. Liu R.. (2020). Gastrointestinal manifestations of SARS-CoV-2 infection and virus load in fecal samples from a Hong Kong cohort: systematic review and meta-analysis. Gastroenterology 159, 81–95. 10.1053/j.gastro.2020.03.065 32251668 PMC7194936

[B14] Coleman J. J. Manavi K. Marson E. J. Botkai A. H. Sapey E. (2020). COVID-19: to be or not to be; that is the diagnostic question. Postgrad. Med. J. 96, 392–398. 10.1136/postgradmedj-2020-137979 32522844 PMC7306267

[B15] Contini C. Di Nuzzo M. Barp N. Bonazza A. De Giorgio R. Tognon M.. (2020). The novel zoonotic COVID-19 pandemic: an expected global health concern. J. Infect. Dev. Ctries 14, 254–264. 10.3855/jidc.12671 32235085

[B16] Cui J. Li F. Shi Z. L. (2019). Origin and evolution of pathogenic coronaviruses. Nat. Rev. Microbiol. 17, 181–192. 10.1038/s41579-018-0118-9 30531947 PMC7097006

[B17] Dantzer R. O'connor J. C. Freund G. G. Johnson R. W. Kelley K. W. (2008). From inflammation to sickness and depression: when the immune system subjugates the brain. Nat. Rev. Neurosci. 9, 46–56. 10.1038/nrn2297 18073775 PMC2919277

[B18] Das G. Mukherjee N. Ghosh S. (2020). Neurological insights of COVID-19 pandemic. ACS Chem. Neurosci. 11, 1206–1209. 10.1021/acschemneuro.0c00201 32320211

[B19] Dowlati Y. Herrmann N. Swardfager W. Liu H. Sham L. Reim E.. (2009). A meta-analysis of cytokines in major depression. Biol. Psychiatry 67, 446–457. 10.1016/j.biopsych.2009.09.033 20015486

[B20] Evrensel A. Ceylan M. E. (2015). The gut-brain axis: the missing link in depression. Clin. Psychopharmacol. Neurosci. 13, 239–244. 10.9758/cpn.2015.13.3.239 26598580 PMC4662178

[B21] Fan H. Tang X. Song Y. Liu P. Chen Y. (2020). Influence of COVID-19 on cerebrovascular disease and its possible mechanism. Neuropsychiatr. Dis. Treat 16, 1359–1367. 10.2147/NDT.S251173 32547039 PMC7266513

[B22] Galland L. (2014). The gut microbiome and the brain. J. Med. Food 17, 1261–1272. 10.1089/jmf.2014.7000 25402818 PMC4259177

[B23] García L. F. (2020). Immune response, inflammation, and the clinical spectrum of COVID-19. Front. Immunol. 11:1441. 10.3389/fimmu.2020.01441 32612615 PMC7308593

[B24] Gunther M. L. Morandi A. Ely E. W. (2008). Pathophysiology of delirium in the intensive care unit. Crit. Care Clin. 24, 45–65. 10.1016/j.ccc.2007.10.002 18241778

[B25] Harapan H. Itoh N. Yufika A. Winardi W. Keam S. Te H.. (2020). Coronavirus disease 2019 (COVID-19): a literature review. J. Infect. Public Health 13, 667–673. 10.1016/j.jiph.2020.03.019 32340833 PMC7142680

[B26] Holshue M. L. Debolt C. Lindquist S. Lofy K. H. Wiesman J. Bruce H.. (2020). First case of 2019 novel coronavirus in the United States. N. Engl. J. Med. 382, 929–936. 10.1056/NEJMoa2001191 32004427 PMC7092802

[B27] Hussain S. Pottathil S. Islam M. Chohan M. Rasool S. (2020). Analysis of codon usage and nucleotide bias in middle east respiratory syndrome coronavirus genes. Evol. Bioinform. 16:117693432091886. 10.1177/1176934320918861 32425493 PMC7218340

[B28] Izzetti R. Nisi M. Gabriele M. Graziani F. (2020). COVID-19 transmission in dental practice: brief review of preventive measures in Italy. J. Dent. Res. 99, 1030–1038. 10.1177/0022034520920580 32302257

[B29] Jiang F. Deng L. Zhang L. Cai Y. Cheung C. W. Xia Z. (2020). Review of the clinical characteristics of coronavirus disease 2019 (COVID-19). J. Gen. Intern. Med. 35, 1545–1549. 10.1007/s11606-020-05762-w 32133578 PMC7088708

[B30] Jiang W. Wu N. Wang X. Chi Y. Zhang Y. Qiu X.. (2015). Dysbiosis gut microbiota associated with inflammation and impaired mucosal immune function in intestine of humans with non-alcoholic fatty liver disease. Sci. Rep. 5:8096. 10.1038/srep08096 25644696 PMC4314632

[B31] Jin X. Lian J. S. Hu J. H. Gao J. Zheng L. Zhang Y. M.. (2020). Epidemiological, clinical, and virological characteristics of 74 cases of coronavirus-infected disease 2019 (COVID-19) with gastrointestinal symptoms. Gut 69, 1002–1009. 10.1136/gutjnl-2020-320926 32213556 PMC7133387

[B32] Kamholz B. (2010). Update on delirium: diagnosis, management, and pathophysiology. Psychiatr. Ann. 40, 52–62. 10.3928/00485718-20091229-05

[B33] Kandeel M. Al-Nazawi M. (2020). Virtual screening and repurposing of FDA approved drugs against COVID-19 main protease. Life Sci. 251:117627. 10.1016/j.lfs.2020.117627 32251634 PMC7194560

[B34] Kannan S. Shaik Syed Ali P. Sheeza A. Hemalatha K. (2020). COVID-19 (Novel Coronavirus 2019)–recent trends. Eur. Rev. Med. Pharmacol. Sci. 24, 2006–2011. 10.26355/eurrev_202002_20378 32141569

[B35] Kotwani A. Gandra S. (2020). Potential pharmacological agents for COVID-19. Indian J. Public Health 64, S112–S116. 10.4103/ijph.IJPH_456_20 32496239

[B36] Lake M. A. (2020). What we know so far: COVID-19 current clinical knowledge and research. Clin. Med. (Lond.) 20, 124–127. 10.7861/clinmed.2019-coron 32139372 PMC7081812

[B37] Li H. Liu S. M. Yu X. H. Tang S. L. Tang C. K. (2020a). Coronavirus disease 2019 (COVID-19): current status and future perspectives. Int. J. Antimicrob. Agents 55:105951. 10.1016/j.ijantimicag.2020.105951 32234466 PMC7139247

[B38] Li Q. Guan X. Wu P. Wang X. Zhou L. Tong Y.. (2020b). Early transmission dynamics in Wuhan, China, of novel coronavirus-infected pneumonia. N. Engl. J. Med. 382, 1199–1207. 10.1056/NEJMoa2001316 31995857 PMC7121484

[B39] Li Y. C. Bai W. Z. Hashikawa T. (2020). The neuroinvasive potential of SARS-CoV2 may play a role in the respiratory failure of COVID-19 patients. J. Med. Virol. 92, 552–555. 10.1002/jmv.25728 32104915 PMC7228394

[B40] Lin L. Jiang X. Zhang Z. Huang S. Zhang Z. Fang Z.. (2020). Gastrointestinal symptoms of 95 cases with SARS-CoV-2 infection. Gut 69, 997–1001. 10.1136/gutjnl-2020-321013 32241899

[B41] Liu Q. Wang R. S. Qu G. Q. Wang Y. Y. Liu P. Zhu Y. Z.. (2020a). Gross examination report of a COVID-19 death autopsy. Fa Yi Xue Za Zhi 36, 21–23. 10.12116/j.issn.1004-5619.2020.01.005 32198987

[B42] Liu W. Wang J. Li W. Zhou Z. Liu S. Rong Z. (2020b). Clinical characteristics of 19 neonates born to mothers with COVID-19. Front. Med. 14, 193–198. 10.1007/s11684-020-0772-y 32285380 PMC7152620

[B43] Liu Y. Gayle A. A. Wilder-Smith A. Rocklöv J. (2020c). The reproductive number of COVID-19 is higher compared to SARS coronavirus. J. Travel Med. 27:taaa021. 10.1093/jtm/taaa021 32052846 PMC7074654

[B44] Lu R. Zhao X. Li J. Niu P. Yang B. Wu H.. (2020). Genomic characterisation and epidemiology of 2019 novel coronavirus: implications for virus origins and receptor binding. Lancet 395, 565–574. 10.1016/S0140-6736(20)30251-8 32007145 PMC7159086

[B45] Macdonald A. Adamis D. Treloar A. Martin F. (2007). C-reactive protein levels predict the incidence of delirium and recovery from it. Age Ageing 36, 222–225. 10.1093/ageing/afl121 17114198

[B46] Maldonado J. R. (2008). Pathoetiological model of delirium: a comprehensive understanding of the neurobiology of delirium and an evidence-based approach to prevention and treatment. Crit. Care Clin. 24, 789–856. 10.1016/j.ccc.2008.06.004 18929943

[B47] McGrane S. Girard T. D. Thompson J. L. Shintani A. K. Woodworth A. Ely E. W.. (2011). Procalcitonin and C-reactive protein levels at admission as predictors of duration of acute brain dysfunction in critically ill patients. Crit. Care 15:R78. 10.1186/cc10070 21366899 PMC3219330

[B48] Memish Z. A. Perlman S. Van Kerkhove M. D. Zumla A. (2020). Middle East respiratory syndrome. Lancet 395, 1063–1077. 10.1016/S0140-6736(19)33221-0 32145185 PMC7155742

[B49] Organization, W. H., and Organization, W. H. (2020). Coronavirus Disease (COVID-2019) Situation Reports. World Health Organization.

[B50] Ozma M. A. Maroufi P. Khodadadi E. Köse S. Esposito I. Ganbarov K.. (2020). Clinical manifestation, diagnosis, prevention, and control of SARS-CoV-2 (COVID-19) during the outbreak period. Infez. Med. 28, 153–165. 32275257

[B51] Pan L. Mu M. Yang P. Sun Y. Wang R. Yan J.. (2020). Clinical characteristics of COVID-19 patients with digestive symptoms in Hubei, China: a descriptive, cross-sectional, multicenter study. Am. J Gastroenterol. 115, 766–773. 10.14309/ajg.0000000000000620 32287140 PMC7172492

[B52] Porfidia A. Pola R. (2020). Venous thromboembolism in COVID-19 patients. J. Thromb. Haemost. 18, 1516–1517. 10.1111/jth.14842 32294289 PMC7262050

[B53] Ressler K. Mayberg H. (2007). Targeting abnormal neural circuits in mood and anxiety disorders: from the laboratory to the clinic. Nat. Neurosci. 10, 1116–1124. 10.1038/nn1944 17726478 PMC2444035

[B54] Rico-Mesa J. S. White A. Anderson A. S. (2020). Outcomes in patients with COVID-19 infection taking ACEI/ARB. Curr. Cardiol. Rep. 22:31. 10.1007/s11886-020-01291-4 32291526 PMC7154066

[B55] Shanmugaraj B. Siriwattananon K. Wangkanont K. Phoolcharoen W. (2020). Perspectives on monoclonal antibody therapy as potential therapeutic intervention for Coronavirus disease-19 (COVID-19). Asian Pac. J. Allergy Immunol. 38, 10–18. 10.12932/AP-200220-0773 32134278

[B56] Shen K. L. Yang Y. H. Jiang R. M. Wang T. Y. Zhao D. C. Jiang Y.. (2020). Updated diagnosis, treatment, and prevention of COVID-19 in children: experts' consensus statement (condensed version of the second edition). World J. Pediatr. 16, 232–239. 10.1007/s12519-020-00362-4 32333248 PMC7180653

[B57] Sifuentes-Rodríguez E. Palacios-Reyes D. (2020). COVID-19: the outbreak caused by a new coronavirus. Bol. Med. Hosp. Infant. Mex. 77, 47–53. 10.24875/BMHIM.20000039 32226003

[B58] Sohrabi C. Alsafi Z. O'neill N. Khan M. Kerwan A. Al-Jabir A.. (2020). World Health Organization declares global emergency: a review of the 2019 novel coronavirus (COVID-19). Int. J. Surg. 76, 71–76. 10.1016/j.ijsu.2020.02.034 32112977 PMC7105032

[B59] Stadler K. Masignani V. Eickmann M. Becker S. Abrignani S. Klenk H. D.. (2003). SARS–beginning to understand a new virus. Nat. Rev. Microbiol. 1, 209–218. 10.1038/nrmicro775 15035025 PMC7097337

[B60] Sternberg E. M. (2006). Neural regulation of innate immunity: a coordinated nonspecific host response to pathogens. Nat. Rev. Immunol. 6, 318–328. 10.1038/nri1810 16557263 PMC1783839

[B61] Tian S. Hu W. Niu L. Liu H. Xu H. Xiao S. Y. (2020). Pulmonary pathology of early-phase 2019 novel coronavirus (COVID-19) pneumonia in two patients with lung cancer. J. Thorac. Oncol. 15, 700–704. 10.1016/j.jtho.2020.02.010 32114094 PMC7128866

[B62] Velavan T. P. Meyer C. G. (2020). The COVID-19 epidemic. Trop. Med. Int. Health 25, 278–280. 10.1111/tmi.13383 32052514 PMC7169770

[B63] Walls A. C. Park Y. J. Tortorici M. A. Wall A. Mcguire A. T. Veesler D. (2020). Structure, function, and antigenicity of the SARS-CoV-2 Spike glycoprotein. Cell 181, 281–292. 10.1016/j.cell.2020.02.058 32155444 PMC7102599

[B64] Wang D. Hu B. Hu C. Zhu F. Liu X. Zhang J.. (2020a). Clinical characteristics of 138 hospitalized patients with 2019 novel coronavirus-infected pneumonia in Wuhan, China. JAMA 323, 1061–1069. 10.1001/jama.2020.1585 32031570 PMC7042881

[B65] Wang Y. Wang Y. Chen Y. Qin Q. (2020b). Unique epidemiological and clinical features of the emerging 2019 novel coronavirus pneumonia (COVID-19) implicate special control measures. J. Med. Virol. 92, 568–576. 10.1002/jmv.25748 32134116 PMC7228347

[B66] Wong M. L. Inserra A. Lewis M. D. Mastronardi C. A. Leong L. Choo J.. (2016). Inflammasome signaling affects anxiety- and depressive-like behavior and gut microbiome composition. Mol. Psychiatry 21, 797–805. 10.1038/mp.2016.46 27090302 PMC4879188

[B67] Wu F. Zhao S. Yu B. Chen Y. M. Wang W. Song Z. G.. (2020a). A new coronavirus associated with human respiratory disease in China. Nature 579, 265–269. 10.1038/s41586-020-2008-3 32015508 PMC7094943

[B68] Wu J. Song S. Cao H. C. Li L. J. (2020b). Liver diseases in COVID-19: etiology, treatment, and prognosis. World J. Gastroenterol. 26, 2286–2293. 10.3748/wjg.v26.i19.2286 32476793 PMC7243650

[B69] Wu Y. Xu X. Chen Z. Duan J. Hashimoto K. Yang L.. (2020c). Nervous system involvement after infection with COVID-19 and other coronaviruses. Brain Behav. Immun. 87, 18–22. 10.1016/j.bbi.2020.03.031 32240762 PMC7146689

[B70] Xiao H. Zhang Y. Kong D. Li S. Yang N. (2020). The effects of social support on sleep quality of medical staff treating patients with coronavirus disease 2019 (COVID-19) in January and February 2020 in China. Med. Sci. Monit. 26:e923549. 10.12659/MSM.923921 32132521 PMC7075079

[B71] Yang L. Tian D. Liu W. (2020a). Strategies for vaccine development of COVID-19. Sheng Wu Gong Cheng Xue Bao 36, 593–604. 10.13345/j.cjb.200094 32347054

[B72] Yang Y. Lu Q. Liu M. Wang Y. Zhang A. Jalali N. (2020b). Epidemiological and clinical features of the 2019 novel coronavirus outbreak in China. medRxiv 2020.2002.2010.20021675. 10.1101/2020.02.10.20021675

[B73] Ye M. Ren Y. Lv T. (2020). Encephalitis as a clinical manifestation of COVID-19. Brain Behav. Immun. 88, 945–946. 10.1016/j.bbi.2020.04.017 32283294 PMC7146652

[B74] Yu N. Li W. Kang Q. Xiong Z. Wang S. Lin X.. (2020). Clinical features and obstetric and neonatal outcomes of pregnant patients with COVID-19 in Wuhan, China: a retrospective, single-centre, descriptive study. Lancet Infect. Dis. 20, 559–564. 10.1016/S1473-3099(20)30176-6 32220284 PMC7158904

[B75] Zhou P. Yang X. L. Wang X. G. Hu B. Zhang L. Zhang W.. (2020a). A pneumonia outbreak associated with a new coronavirus of probable bat origin. Nature 579, 270–273. 10.1038/s41586-020-2012-7 32015507 PMC7095418

[B76] Zhou Z. Zhao N. Shu Y. Han S. Chen B. Shu X. (2020b). Effect of gastrointestinal symptoms in patients with COVID-19. Gastroenterology 158, 2294–2297 10.1053/j.gastro.2020.03.020 32199880 PMC7270807

